# Supplementary material for: Over a century of global decline in the growth performance of marine fishes
Source: Nat Commun. 2026 Feb 10;17:2612. doi: 10.1038/s41467-026-69416-x (PMC13003017; doi:10.1038/s41467-026-69416-x)
Supplement: Supplementary file 1 — Supplementary Information [file 41467_2026_69416_MOESM1_ESM.pdf]

Supplementary materials for:

Over a century of global decline in the growth performance of marine fishes

Nature Communications

Helen F. Yan[1,2]*, Hannah V. Watkins[3], Alexandre C. Siqueira[1,4], David R. Bellwood[1]

[1]*Research Hub for Coral Reef Ecosystem Functions, College of Science and Engineering, James Cook University, Townsville, QLD 4811, Australia*
[2]*Thriving Oceans Research Hub, School of Geosciences, University of Sydney, Camperdown, NSW 2006, Australia*
[3]*School of Resource and Environmental Management, Simon Fraser University, Burnaby, BC, V5A 1S6, Canada*
[4]*Centre for Marine Ecosystems Research, School of Science, Edith Cowan University, Perth, WA 6027, Australia*

*Corresponding author: Helen F. Yan, *Thriving Oceans Research Hub, University of Sydney*, Camperdown, NSW 2006, Australia.
Email: helen.yan@sydney.edu.au

Supplementary methods

Varying the slope between $\log_{10}K$ and $\log_{10}L_\infty$ to generate empirically measured S_L values resulted in a distribution of growth performance that produced negative values (Fig. S8), which is typically outside the range of expected growth performance values. Consequently, we used a similar autoregressive state-space model but specified a gaussian distribution.

$$\text{Normal}\left(y_{i,t}, \sigma\right) \tag{S1}$$

$$y_{i,t} = \alpha_t + \alpha_{fam} + \beta X_i \tag{S2}$$

$$\sigma \sim \text{Student-t}(3, 0, 2.5) \tag{S3}$$

Here, y_i is the growth performance of fishes in year t following a normal error distribution with scale (standard deviation) σ. As in Equation 14 in the main text, the term α_t is the expected mean growth performance for each year for each aging method, α_{fam} is the varying effect of family on the growth performance of fishes. The term β is a vector of estimated population-level effects and X is the extracted temperature for each specified location for each growth performance value y_i. The rest of the model was specified following equations 11-23.

Supplementary figures

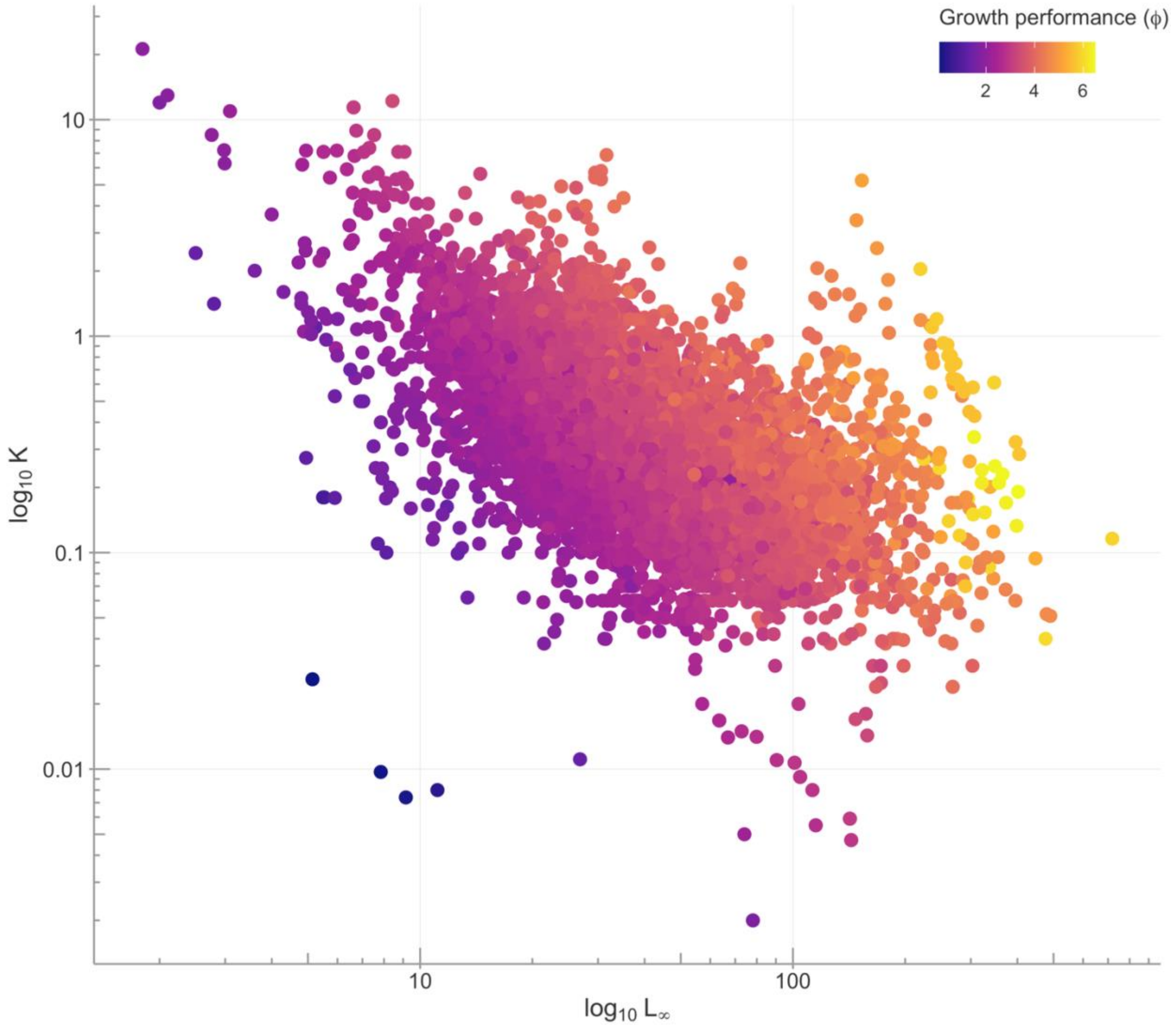

Figure S1. Growth performance of marine fishes. Each point is representative of a single population/stock and is illustrating the mined growth parameters L_∞ and K, shown on the x and y axes, respectively (n = 7683). Points are coloured based on the calculated growth performance value (ϕ), whereby the lowest values are in purple and the highest are in yellow. Note both x and y axes are on the $\log_{10}$ scale. Source data are provided as a Source Data file.

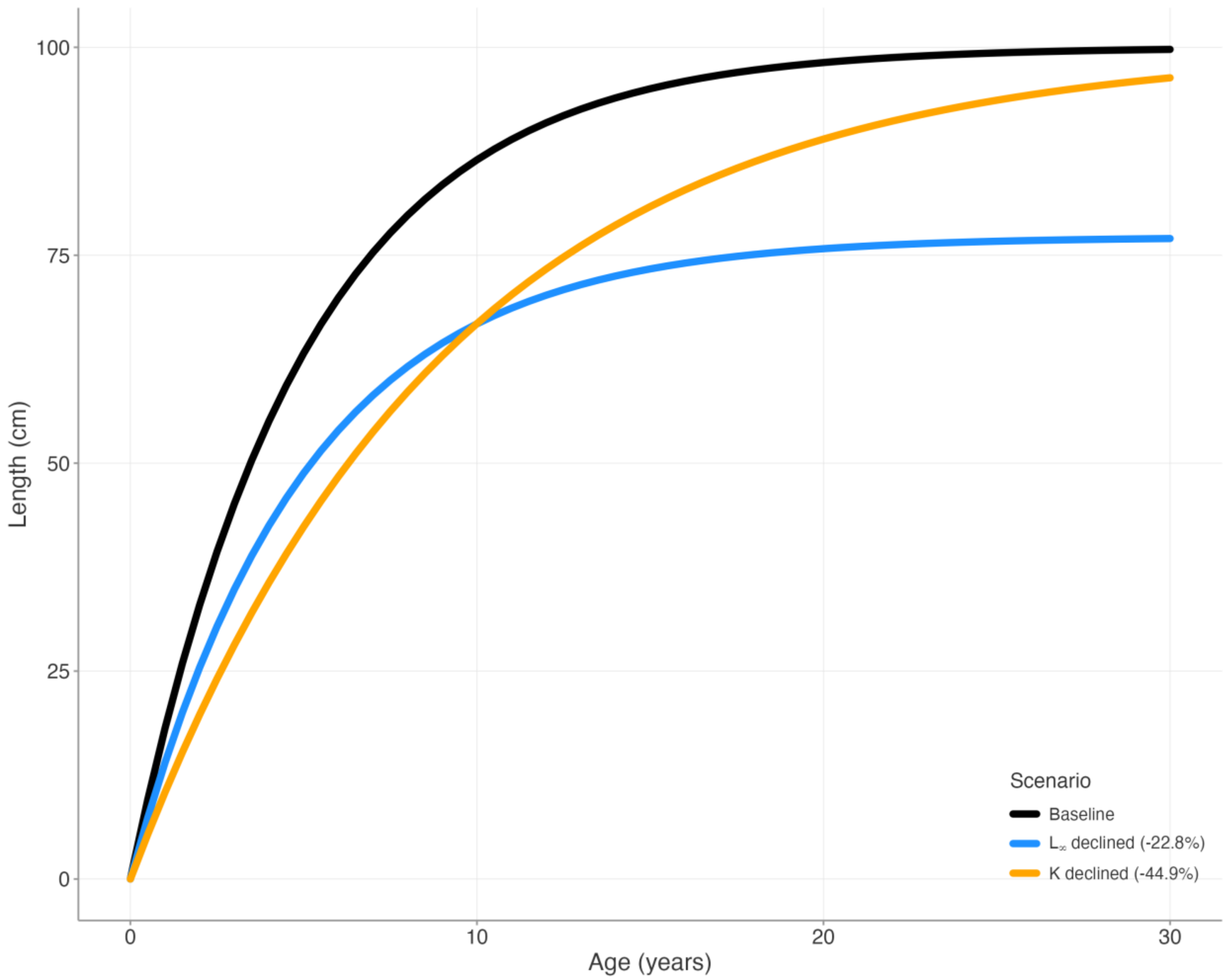

Figure S2. Changes in the von Bertalanffy growth curve with changing L_∞ and *K*. Simulation showing how the baseline von Bertalanffy growth curve (black) changes when L_∞ declines by 22.8% while holding *K* constant (blue), and when *K* declines by 44.9% while holding L_∞ constant (orange; see Methods). Source data are provided as a Source Data file.

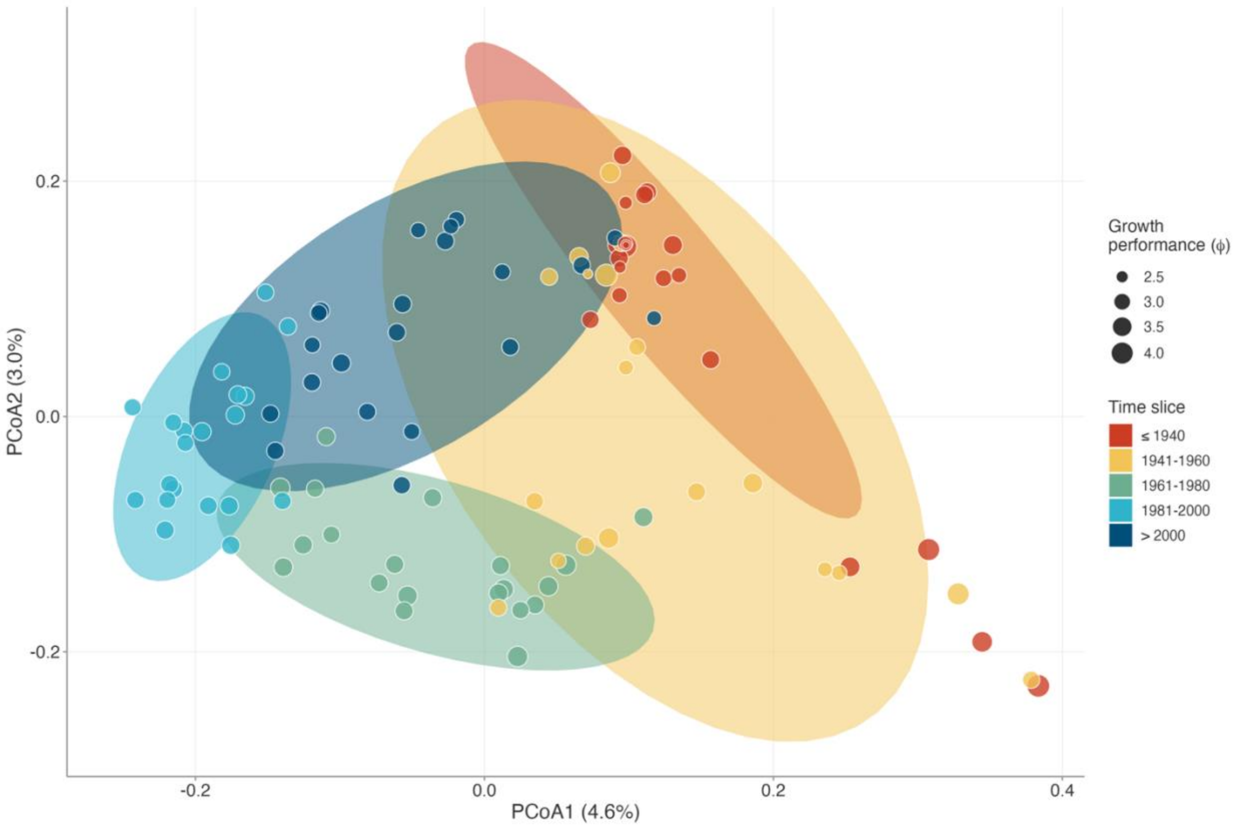

Figure S3. Principal Coordinate Analysis (PCoA) of species compositional changes in growth studies through time. Each point is representative of the species composition for a given year based on presence/absence and are size based on the median growth performance value per year. Ellipses show the 90% confidence spread in the data. Points and ellipses are coloured based on their respective 20-year time slices: prior to 1940 (inclusive; red; *n* = 43 species), 1941-1960 (yellow; *n* = 260), 1961-1980 (green; *n* = 914), 1981-2000 (light blue; *n* = 2154), and after 2000 (dark blue; *n* = 1146). The variance explained by each axis is shown in parentheses. Source data are provided as a Source Data file.

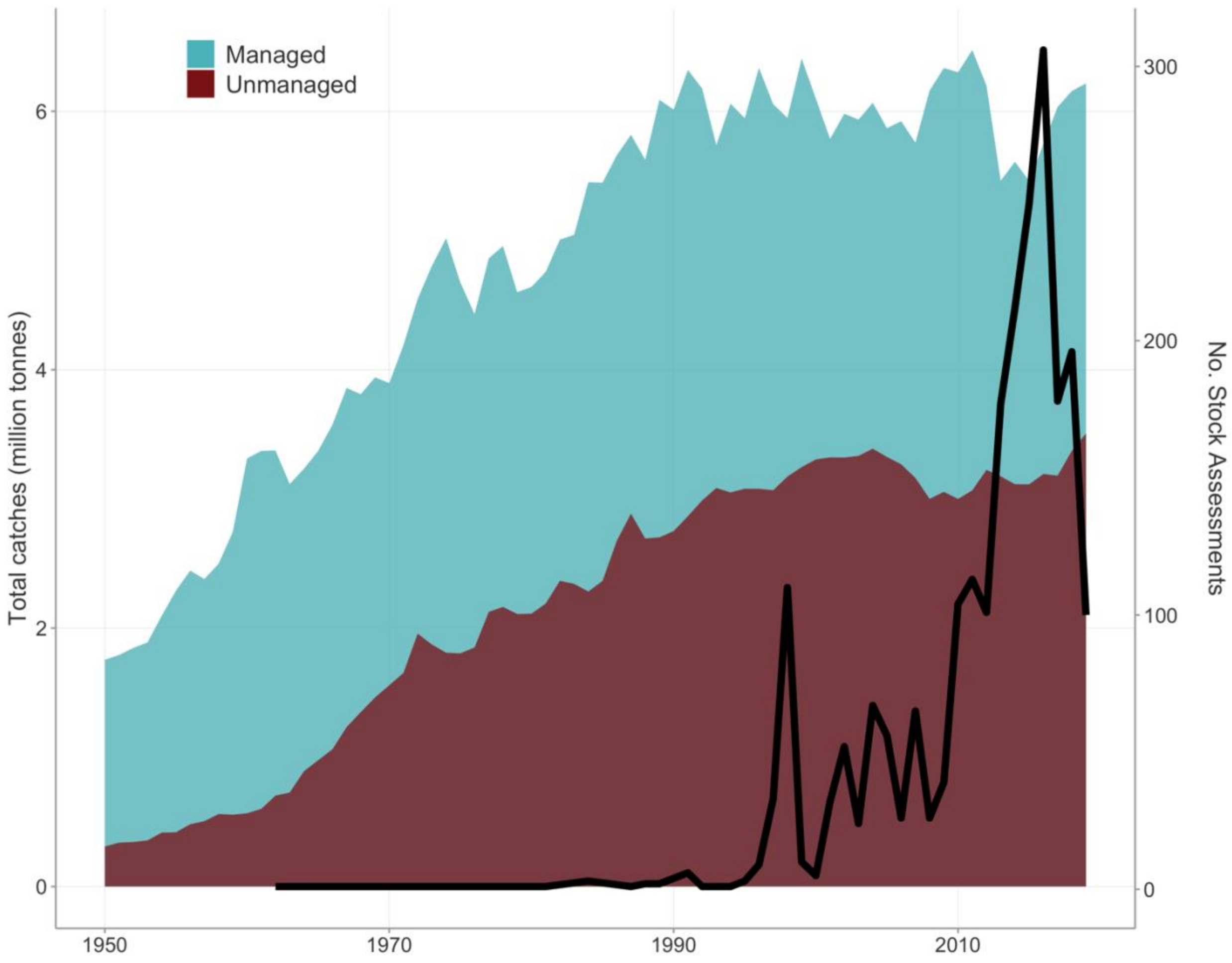

Figure S4. Temporal mismatch between fishing and management. The total reported and reconstructed landed tonnes of managed fishes (blue) and unmanaged fishes (red) from 1950 to 2021 (left y axis) across all countries. Note the polygons are overlapping. The black line denotes the number of stock assessments in a given year (right y axis) across the time series. Source data are provided as a Source Data file.

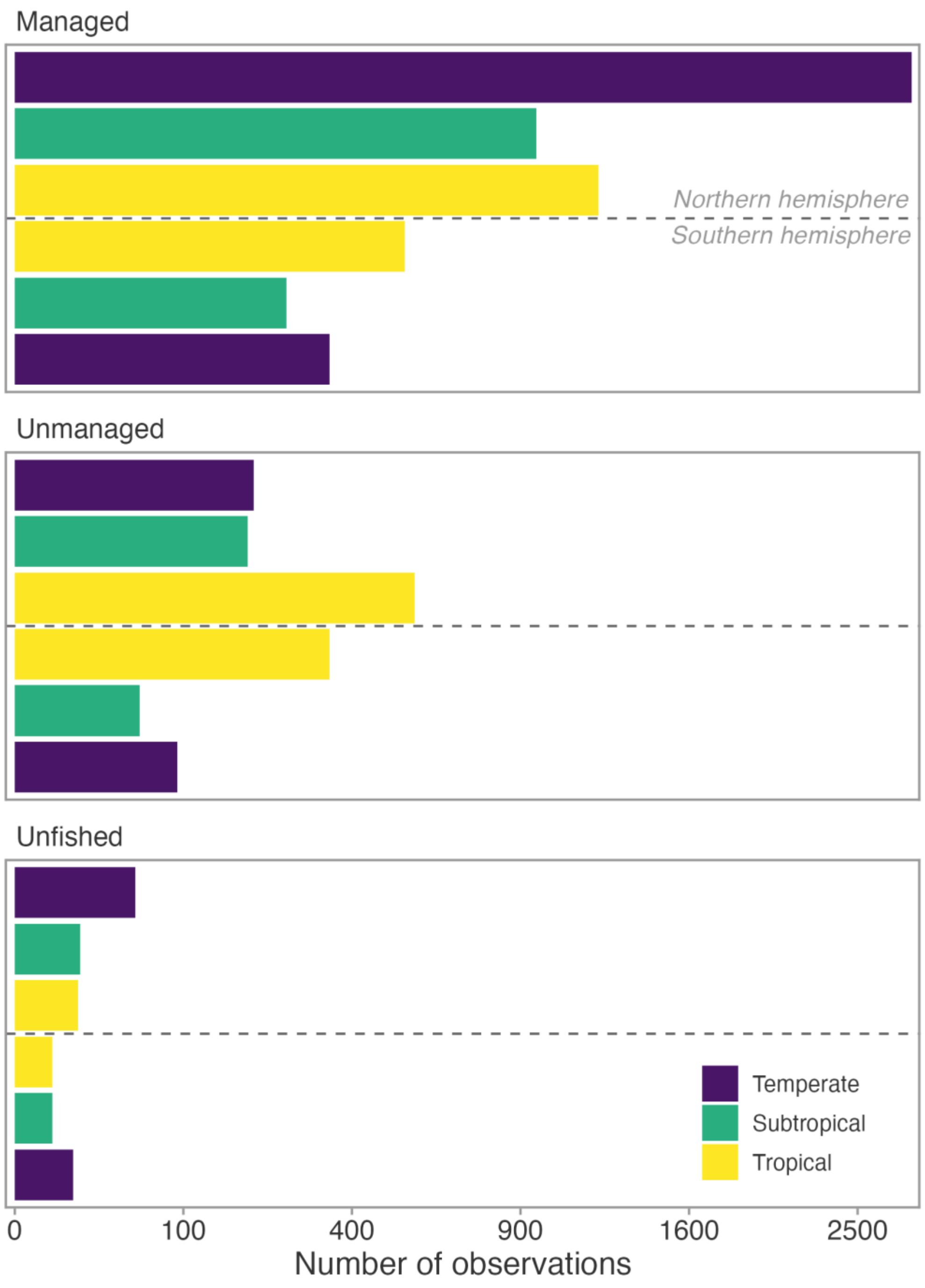

Figure S5. Total number of studies by region and management. Reformatting of Figure 4 of the main text showing the number of observations in managed fisheries (top), unmanaged fisheries (middle), unfished species (bottom) in temperate (purple), subtropical (green), and tropical locations (yellow). Note that this version of the figure has used a square root transformation on the x axis. Source data are provided as a Source Data file.

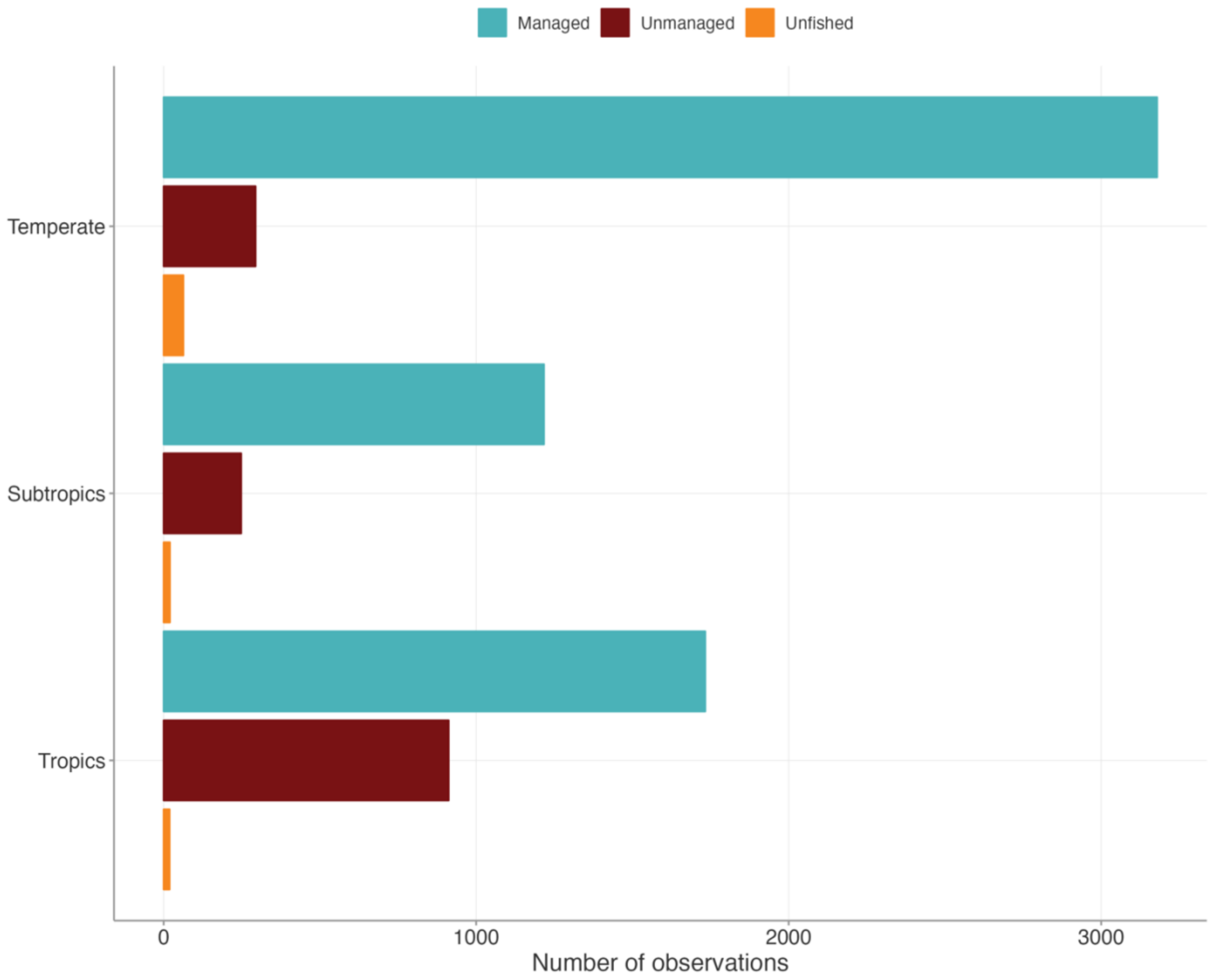

Figure S6. Dominance of managed fisheries in temperate regions. Total number of observations in the study from managed fisheries (green), unmanaged fisheries (red), and unfished species (orange) in temperate (top), subtropical (middle), and tropical regions (bottom). Source data are provided as a Source Data file.

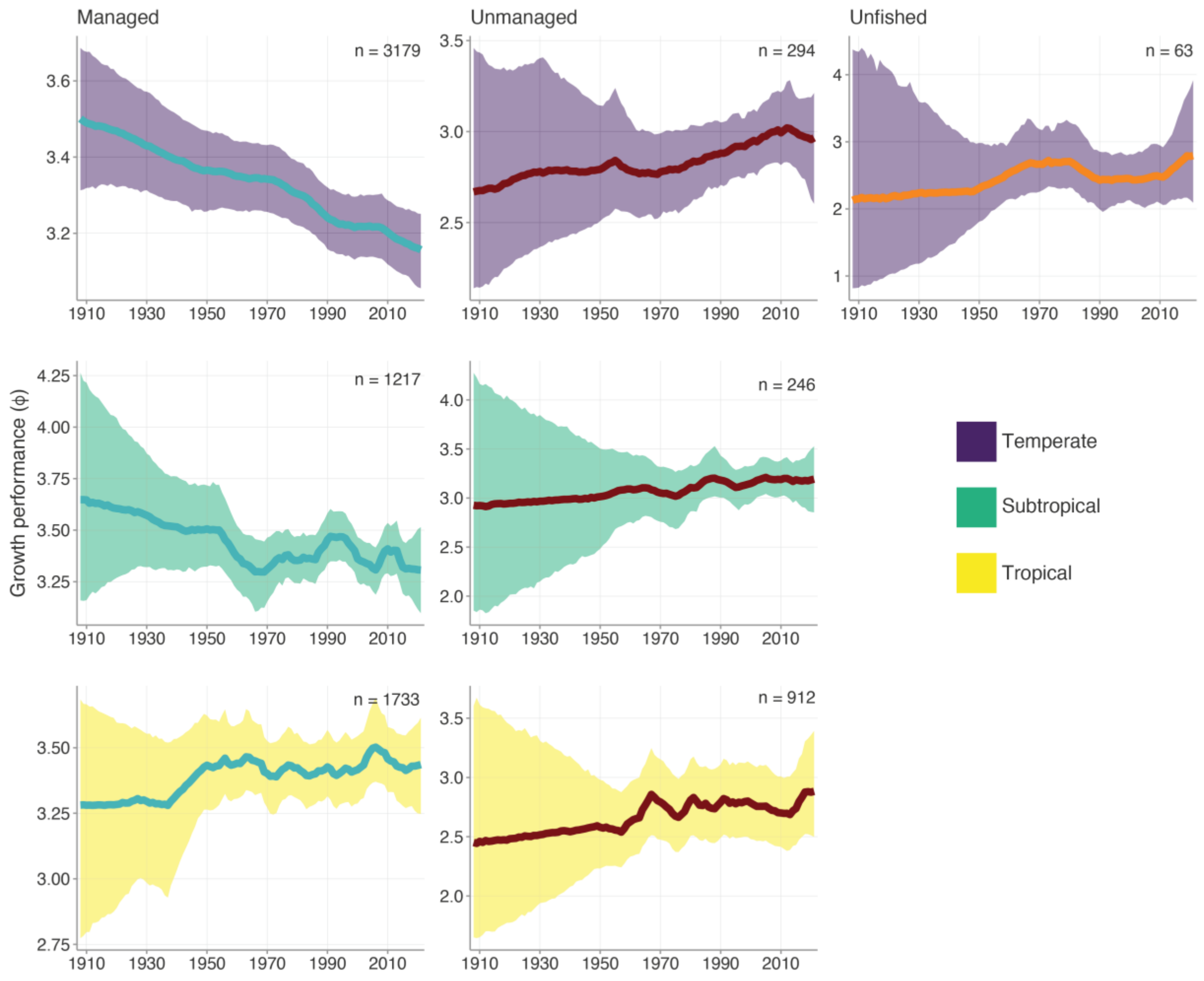

Figure S7. Changes in growth performance of fishes across regions and management schemes. Changes in growth performance (ϕ) of fishes in temperate (top row), subtropical (middle row), and tropical regions (bottom row) for managed fishes (left column), unmanaged fishes (middle column), and unfished species (right). The thick ribbons denote the 90% credible intervals and the thick lines are the median estimates. Note trends could not be estimated for subtropical and tropical unfished species due to an insufficient number of observations (n = 20 and 19, respectively), and all y axes are on different scales. Source data are provided as a Source Data file.

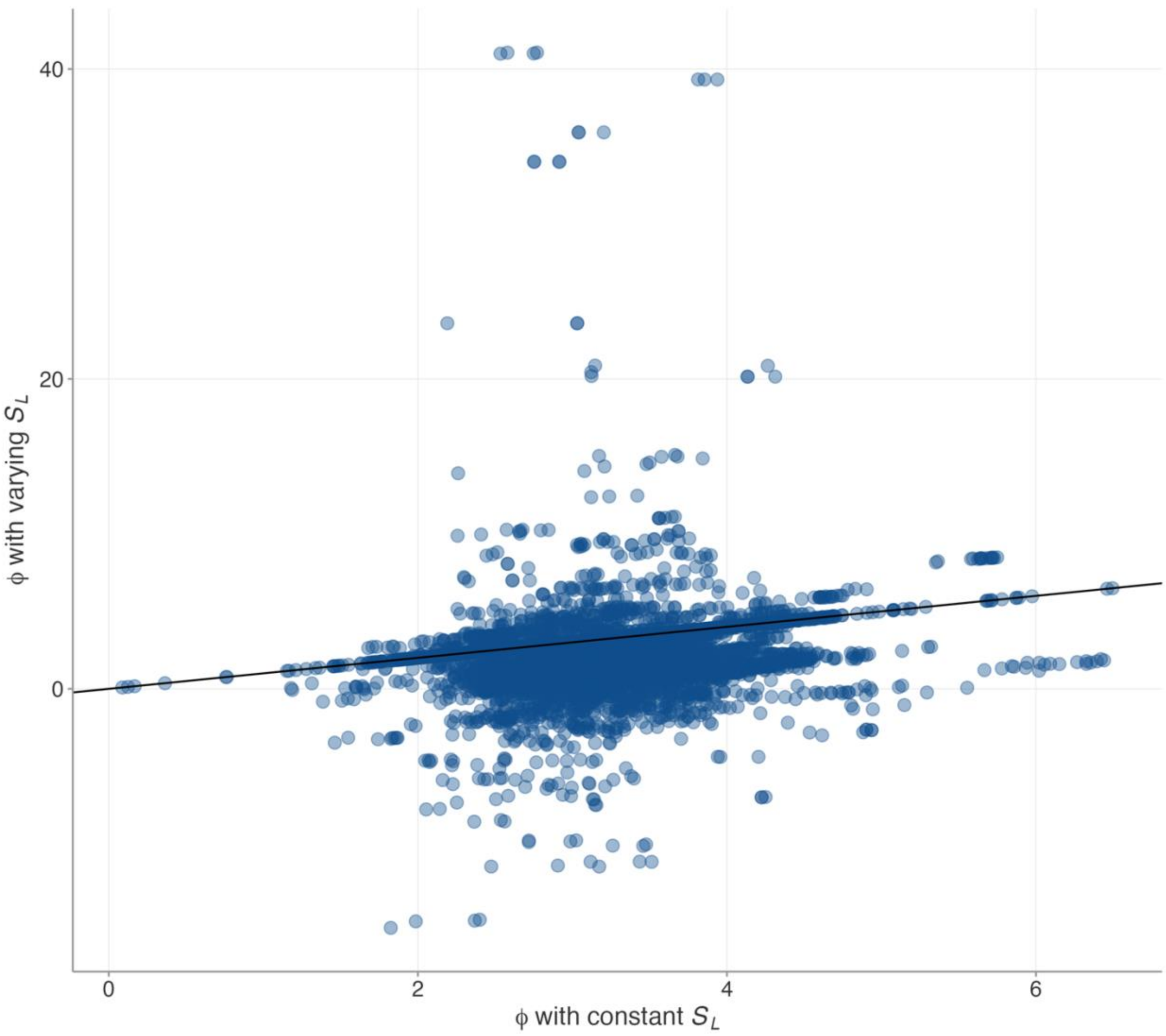

Figure S8. Variation in growth performance (ϕ) by varying S_L. Comparison of growth performance using an average S_L value calculated from the constant mean anabolic term for fishes and the length-weight regression exponent (see Equations 2-3 in the Methods) on the x axis compared to growth performance calculated from empirically measured S_L values on the y axis. The black line is the 1:1 line. Source data are provided as a Source Data file.

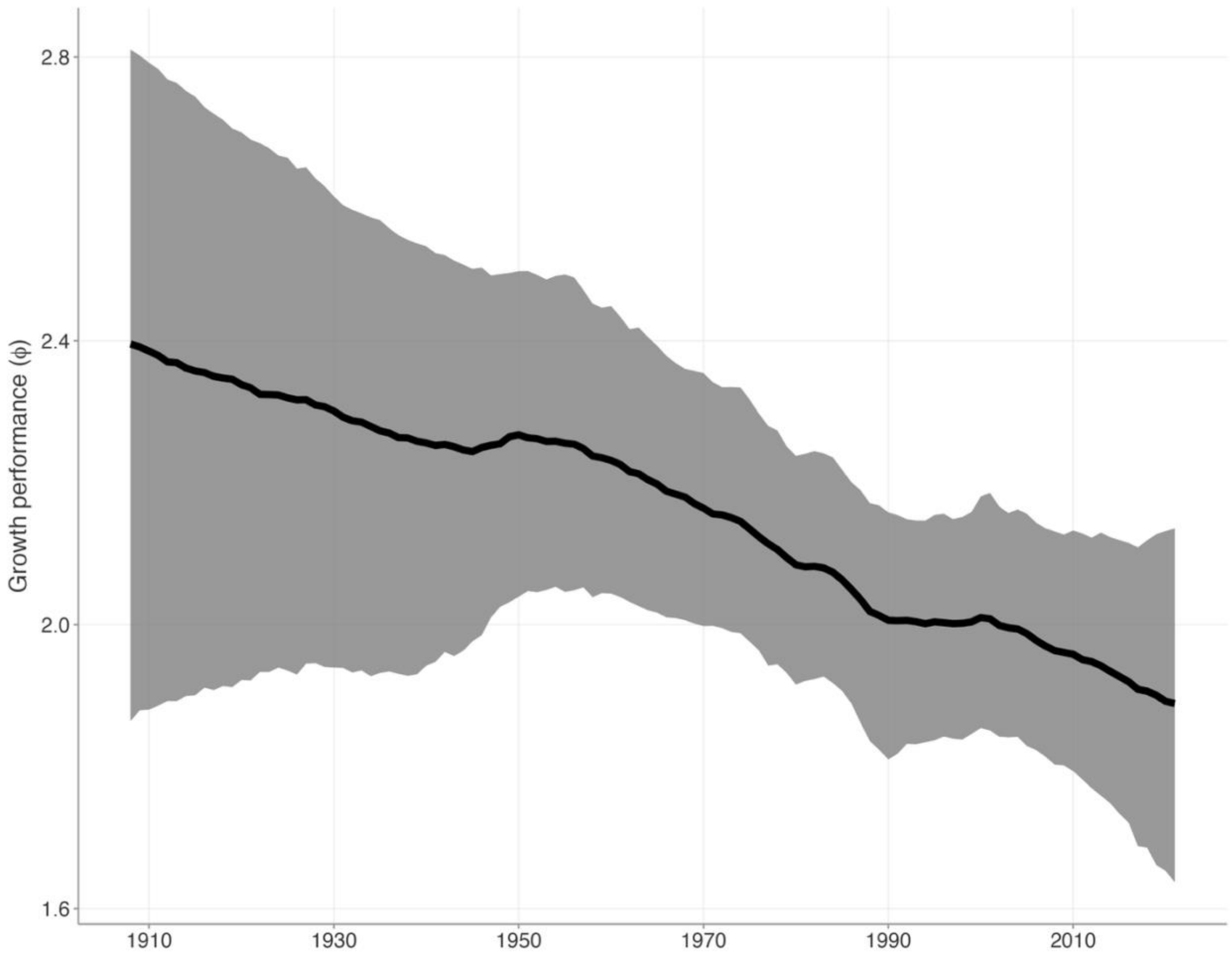

Figure S9. Global time series using varying S_L. Changes in global growth performance of marine fishes with empirically measured S_L values (n = 7683). The grey ribbon is the 90% credible interval and the thick black line is the median modelled trend. Source data are provided as a Source Data file.

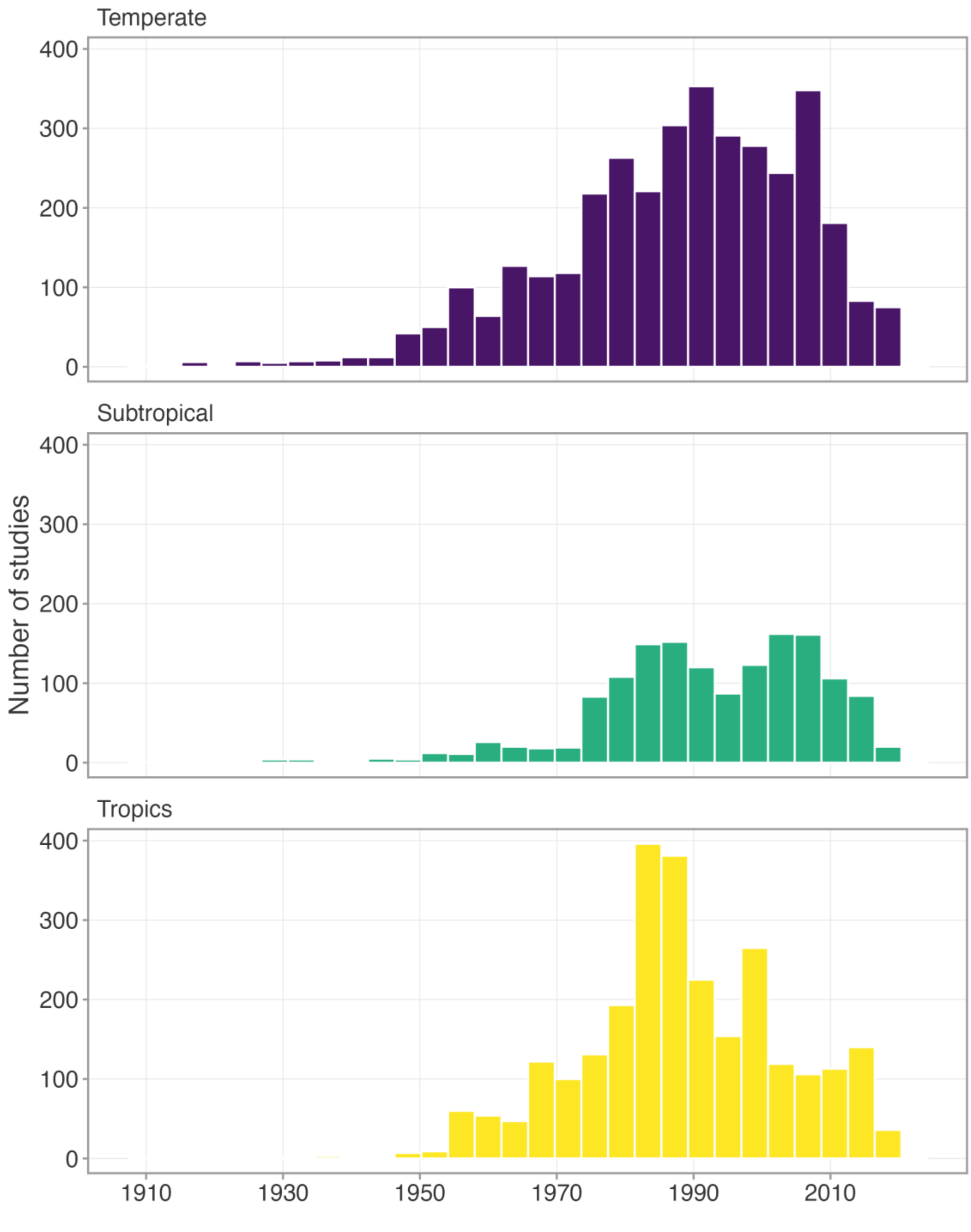

Figure S10. Distribution of regional studies through time. Number of growth studies in temperate (purple, *n* = 3536), subtropical (green, *n* = 1483) and tropical (yellow, *n* = 2664) locations per year. Source data are provided as a Source Data file.
