# Supplementary material for: Over a century of global decline in the growth performance of marine fishes
Source: Nat Commun. 2026 Feb 10;17:2612. doi: 10.1038/s41467-026-69416-x (PMC13003017; doi:10.1038/s41467-026-69416-x)
Supplement: Supplementary file 3 — Description of Additional Supplementary Files [file 41467_2026_69416_MOESM3_ESM.pdf]

Description of Additional Supplementary Files

File Name: Supplementary Data 1
Description: **Model diagnostic values.**
Posterior medians, lower and upper 90% credible intervals, effective sample size (ESS), and Rhat values for estimated coefficients from the global, managed, unmanaged, unfished, temperate, subtropical, and tropical models (see Methods for coefficient definitions).
